# Predictive Levels of CD24 in Peripheral Blood Leukocytes for the Early Detection of Colorectal Adenomas and Adenocarcinomas

**DOI:** 10.1155/2015/916098

**Published:** 2015-05-11

**Authors:** Sarah Kraus, Shiran Shapira, Dina Kazanov, Inna Naumov, Menachem Moshkowitz, Erwin Santo, Lior Galazan, Ravit Geva, Einat Shmueli, Aharon Hallack, Nadir Arber

**Affiliations:** ^1^The Integrated Cancer Prevention Center, Tel Aviv Sourasky Medical Center, Tel Aviv University, Tel Aviv, Israel; ^2^Sackler School of Medicine, Tel Aviv University, Tel Aviv, Israel; ^3^Department of Gastroenterology, Tel Aviv Sourasky Medical Center, Tel Aviv University, Tel Aviv, Israel; ^4^Department of Oncology, Tel Aviv Sourasky Medical Center, Tel Aviv University, Tel Aviv, Israel

## Abstract

CD24 is expressed in 90% of colorectal adenomas and adenocarcinomas. Colorectal cancer (CRC) can be mostly prevented but average risk population screening by stool testing or colonoscopy faces many hurdles. Blood testing is clinically needed. We aimed to evaluate the utility of CD24 expression in peripheral blood leukocytes (PBLs). Two independent case studies were conducted in eligible individuals undergoing colonoscopy. Protein extracted from PBLs was subjected to immunoblotting using anti-CD24 monoclonal antibodies. CD24 sensitivity and specificity were determined using receiver operating characteristic (ROC) analysis. Initially, 150 subjects were examined: 63 had CRC, 19 had adenomas, and 68 had normal colonoscopies. The sensitivity and specificity of CD24 for distinguishing CRC from normal subjects were 70.5% (95% CI, 54.8–83.2%) and 83.8% (95% CI, 74.6–92.7%) and for adenomas 84.2% (95% CI, 60.4–96.4%) and 73.5% (95% CI, 61.4–83.5%), respectively. In the second trial (*n* = 149), a similar specificity but higher sensitivity was achieved: 80.0% (95% CI, 63.1–91.6%) for CRC and 89.2% (95% CI, 74.6–97%) for adenomas. A simple noninvasive blood test evaluating CD24 levels has high sensitivity and specificity for detecting colorectal adenomas and cancer in patients undergoing colonoscopy at an urban medical center. Larger multicenter studies are warranted to establish the potential of this promising test.

## 1. Background

Colorectal cancer (CRC) is a major health concern worldwide that typically develops over many years from normal appearing mucosa through its precursor lesion, the adenomatous polyp, providing ample opportunities for early detection and intervention [1, 2]. Early diagnosis of CRC has been shown to improve prognosis and in turn decrease disease-associated morbidity and mortality. A number of screening modalities are recommended for adenoma and CRC detection, each with related advantages and disadvantages that impact patient's acceptance and compliance, which are generally low [3, 4].

A simple, noninvasive test that could reliably identify individuals with colorectal adenomas or early carcinoma not only would have great utility for CRC early detection, but also will be able to prevent the disease and be more widely accepted by the general population. Of the current modalities available for CRC screening, colonoscopy and fecal occult blood testing are most often recommended. While colonoscopy is considered the “golden standard” for CRC screening, it is expensive and invasive and carries a number of risks including bleeding and perforation. Patient acceptance is variable due to these factors and also because of the tedious bowel preparation and anticipated procedure-related pain/discomfort and embarrassment. Stool testing, although noninvasive, is limited by low sensitivity particularly for adenomas and has poor compliance since it requires annual collection which is often incomplete.

A simple blood test would increase screening compliance, promoting early detection and better patient outcomes. Such an example is the blood-based Septin 9 (SEPT9) methylated DNA test which specifically detects CRCs with an overall sensitivity of 90% [5, 6]. However, the plasma Septin 9 test detected only 12% of adenomas with a false-positive rate of 3% and the stool test was later shown to be more accurate [7].

The need for a noninvasive test has led investigators to explore the use of gene expression microarrays and serum proteomics for the detection of CRC and adenomas.

Using gene expression array, we have previously demonstrated that CD24, a mucin-like glycosylphosphatidylinositol- (GPI-) anchored protein, is differentially expressed in normal and transformed enterocytes and that its overexpression in malignant cells reverts to normal following cyclooxygenase-2 (COX-2) inhibition [8–10]. CD24 consists of a small protein core, comprising 27 amino acids, which is extensively glycosylated. Its final molecular weight varies between 28 and 75 kDa [10, 11]. Immunohistochemical analysis of human colonic specimens showed differential staining patterns for CD24 in normal tissue, colonic adenomas, and adenocarcinomas. CD24 expression was detected in 90.7% of colorectal adenomas and 86.3% of CRCs compared to weak expression in only 16% of adjacent normal epithelium [10]. The overexpression of CD24 during CRC progression and its downregulation by COX-2 inhibition suggests a significant role in the oncogenic pathway involved in CRC carcinogenesis.

Human* CD24* mRNA has a 0.24-kb ORF and a 1.8-kb untranslated region (UTR). Four* CD24* genetic variants have been described, a C to T single nucleotide polymorphism (SNP) at position 170 from the CD24 translation start site (P170) leading to an alanine by valine exchange in codon 57 (A57V) and three other polymorphic sites located at the 3′-UTR, P1056 A/G, P1527 TG/*del*, and P1626 A/G. These SNPs have been associated with an increased risk and a more rapid progression of multiple sclerosis and other autoimmune diseases and may possess functional relevance, thereby affecting CD24 protein levels and mRNA stability [12, 13].

In the current study, we demonstrate that CD24 level in peripheral blood leukocytes (PBLs) can be used as a potential marker for the detection of CR neoplasia and examine the possible implications of* CD24* genetic variants in the genetic predisposition to CRC. We demonstrate that anti-CD24 monoclonal antibodies recognize CD24 expressed in PBLs isolated from plasma and compare its levels in patients undergoing colonoscopy who have CR adenomas, adenocarcinomas, or normal colon. Herein, we show that CD24 expression in PBLs can serve as a potential promising screening tool to select which healthy subjects are in fact at risk of having CR neoplasia and need to undergo screening colonoscopy.

## 2. Materials and Methods

### 2.1. Subjects

During 2009 to 2011, blood samples were obtained from consented individuals older than 40 years of age presenting for colonoscopy at the Integrated Cancer Prevention Center and patients with either known or suspected colorectal neoplasia, from the Departments of Oncology and Gastroenterology at Tel Aviv Sourasky Medical Center. Persons with a known family history of CRC or polyposis syndromes, inflammatory bowel disease, or other cancers were excluded. Eligible subjects completed a detailed medical questionnaire including cancer diagnoses, demographic data, and other epidemiologic information. Each participant underwent a physical examination and blood sampling prior to the colonoscopic evaluation. Blood specimens were drawn using a standard operating procedure, so that collection and handling would occur uniformly. Patient information was deidentified and only anonymized data was available to the investigators.

All tissue specimens obtained at the time of colonoscopy were sent to a central pathology laboratory. Polyps were classified as hyperplastic, adenomatous (±villous or high-grade features), or other (i.e., normal mucosa, lymphoid aggregate, and inflammatory polyp). Subjects were thereafter classified based on the colonoscopic and histologic findings as normal/hyperplastic polyp(s), adenoma(s), and advanced adenoma(s). An advanced adenoma was defined as an adenomatous polyp with any of the following features: ≥1 centimeter, containing villous features, or displaying high-grade dysplasia. Size of polyp was determined by the endoscopist and not based on the pathology specimen analyzed. Subjects with multiple adenomas were classified according to the largest polyp size.

Written informed consent was obtained from all eligible participants prior to entry into the study. The study was approved by the Institutional Review Board of Tel Aviv Sourasky Medical Center and the Israeli Ministry of Health.

Blood samples from two independent case-study cohorts were analyzed. Samples from the first cohort were also analyzed by a blinded investigator at the Cancer and Vascular Biology Research Center (Technion, Haifa, Israel). In the second independent cohort comprised of new patients, those with high levels of C-reactive protein (CRP) (>5 mg/L) and/or white blood cell (WBC) counts (>10.9 × 10^3^/*μ*L) were excluded from analysis, based on previous data suggesting a possible association between CD24 levels and inflammation (unpublished observations).

### 2.2. Isolation of Peripheral Blood Leukocytes and Western Blot Analysis

Blood was collected into 9-mL collection tubes (Vacuette, Greiner Bio-One). All samples were collected and processed identically. PBLs were isolated from blood samples by collecting buffy coats obtained after centrifugation for 3 minutes at 3000 rpm and discarding the plasma supernatant. Residual erythrocytes were lysed by brief incubation in erythrocyte lysis buffer (ELB) containing 155 mM NH_4_Cl, 0.1 mM EDTA, and 10 mM KHCO_3_. The resulting pellet was washed in ELB and lysed (20 minutes on ice) in the presence of 1% Triton X-100 containing a protease inhibitor cocktail (Roche, Germany) and centrifuged at 15,000 g for 15 minutes at 4°C. The protein concentration in the lysates was determined using the Bio-Rad protein assay (Bio-Rad, Germany) and protein extracts (20 *μ*g) were subjected to SDS-PAGE and Western blotting using a highly specific anti-CD24 SWA11 monoclonal antibody (mAb) [14, 15]. No contaminating protein bands were detected in any of the CD24 immunoblots. Each sample was analyzed twice by two different investigators (Sarah Kraus, Dina Kazanov) and normalized against actin as a housekeeping loading control to ensure that protein loading was uniform. Band intensities were quantitated by densitometry using the imaging TINA 2.0 software. The relative intensity was expressed in optical density (arbitrary units) per unit area (OD/mm^2^).

### 2.3. CD24 Polymorphism Genotyping

DNA was obtained from peripheral blood, using standard methods. Samples were genotyped using PCR amplification and restriction fragment length polymorphism (RFLP) analysis. DNA fragments bearing the* CD24* 170 C/T polymorphic site (P170; rs8734), in the coding region of exon 2 (GenBank accession number NM_013230), were amplified by PCR using a forward primer (5′-TTGTTGCCACTTGGCATTTTTGAGGC-3′) and a reverse primer (5′-GGATTGGGTTTAGAAGATGGGGAAA-3′). The predicted* CD24* PCR fragment is 453 bp long. The T→C change yields a* Bst*XI restriction enzyme site enabling us to differentiate the two different* CD24* alleles. For the 1527~1528 TG/*del* (P1527; rs3838646), P1056 A/G (rs1058818), and P1626 A/G (rs1058881) polymorphisms, the functional* CD24* locus was selectively amplified by nested PCR. The first PCR amplification was from intron 1 to the end of exon 2 by using a forward primer (5′-CTAAAGAGAATGACCTTGGTGGGT TGA G-3′) and a reverse primer (5′-CACAGTAGCTTCAAAACTGTTCGA-3′). The predicted* CD24* PCR fragment is 2,017 bp long. The second PCR amplification was based on the polymorphic site using a forward primer (5′-GCAATTTTGCCTTCAAAACAG-3′) and a reverse primer (5′-TTTAGG CTT AGGACCAGGTTC-3′) for P1527, a forward primer (5′-AATCTACCCCCAGAT CCAAGCA-3′) and a reverse primer (5′-GCAATTTTGCCTTCAAAACAG-3′) for P1056, and a forward primer (5′-CAACTATGGATCAGAATAGCAACAAT-3′) and a reverse primer (5′-GGAACATCTAAGCATCAGTGTGTG-3′) for P1626. The PCR products were digested overnight with BsrI (65°C) for P1527, BstUI (60°C) for P1056, and MfeI (37°C) for P1626 (New England Biolabs, Inc.) and then electrophoresed on 2.0% Agarose gels. The validity of the PCR-RFLP analysis was confirmed by direct sequencing of several PCR samples with each genotype.

### 2.4. Statistical Analysis

The densitometry results were analyzed using MedCalc statistical software. Receiver operating characteristic (ROC) curves were created to assess the performance of the CD24 test in detecting CR adenomas and adenocarcinomas [16]. The cutoff value was set for each experiment independently, in order to get the best sensitivity and specificity and due to the fact that normalization standards do not yet exist. The CD24 results were dichotomized based on whether they were above or below the cutoff level and the sensitivity and specificity (with 95% confidence intervals (CI)), and positive and negative predictive values for CD24 were determined. Discrimination of the CD24 test between patients with levels above and below the threshold CD24 value was quantified by the area under the ROC curve (AUC) and reported with 95% CI.

Univariate analysis was performed using SAS statistical software (version 9.1; SAS Institute, Inc.) to assess the relationship between CD24 expression and potential predictive variables related to clinicopathologic features, including gender, age, presence of advanced adenoma (≥1 cm), and CRC stage. Categorical outcome data was reported as frequencies. Comparisons between those subjects with CD24 above/below the cutoff and the aforementioned variables were performed using Fisher's exact test. Continuous data was reported in mean values and compared using Student's *t*-test. A two-sided *p* value of <0.05 was considered statistically significant. Only variables attaining statistical significance in the univariate analysis would be included in a multivariable logistic regression analysis.

Analysis of the* CD24* polymorphism data was performed with SPSS software (version 12.0). The associations between* CD24* polymorphism carriers and CD24 protein levels were analyzed by Student's *t*-test for independent samples. Confidence intervals are all reported at the 95% significance level, and all *p* values are two-sided (*α* = 0.05). Multiple logistic regression analysis was used to calculate the risk of colorectal neoplasia. Multiplicative interactions were evaluated using an interaction term; significance to the logistic model was determined by the log rank test. Database organization and management were performed in Microsoft Excel (Redmond, WA).

## 3. Results

**Figure 1:**
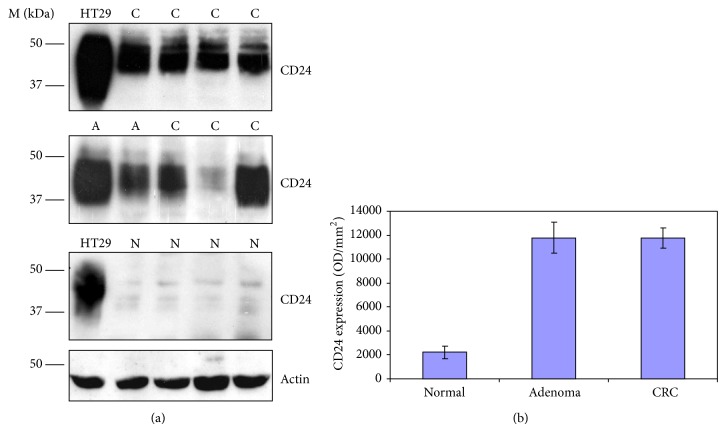
Expression of CD24 in peripheral blood cell lysates from adenoma, CRC patients, and healthy subjects. (a) Samples (20 *μ*g/lane) from adenoma (A), CRC (C) patients, and normal subjects (N) were subjected to SDS-PAGE and Western blotting using anti-CD24 mAb (SWA11). M depicts molecular weight markers. (b) CD24 expression levels in samples from the exploratory study as determined by densitometry (TINA 2.0). Expression levels are presented as optical density (arbitrary units) per unit area (OD/mm^2^). The results in the bar graph represent the median values ± S.E.

In the first case-study cohort data from 150 eligible participants was analyzed (Table 1). Colonoscopy and histologic evaluation revealed the following among subjects: 63/150 (42%) with CRC, 19/150 (13%) with at least one adenoma, and 68/150 (45%) with normal colonoscopy. Western blot analysis showed high level of CD24 expression in PBLs obtained from individuals with adenomas and adenocarcinomas compared to those with normal colonoscopies. The median values for CD24 protein level were 11773 ± 846 (OD/mm^2^  ±  S.E.) in CRC patients, 11771 ± 1287 (OD/mm^2^  ±  S.E.) in patients with adenomas, and 2227 ± 535 (OD/mm^2^  ±  S.E.) in normal subjects (*p* < 0.001) (Figures 1(a) and 1(b)). The median values for CD24 expression in lymphoid aggregates and inflammatory polyps were very low, 929 ± 366 (OD/mm^2^  ±  S.E.).

**Figure 2:**
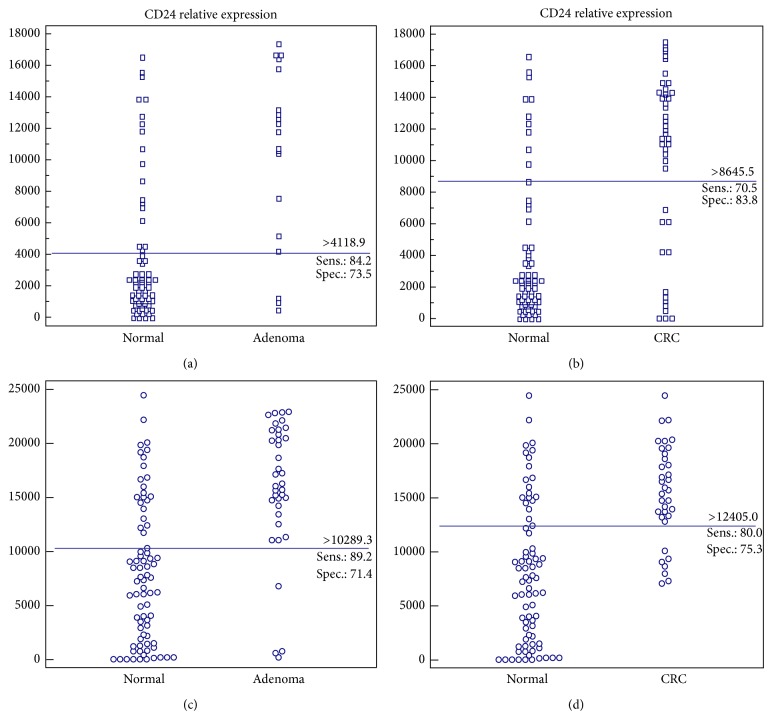
Distribution of patients with adenoma and CRC compared to normal. CD24 levels in subjects with adenoma and CRC compared to normal. Dot diagrams represent the relative expression of CD24 as determined by densitometry and expressed in optical density (arbitrary units) per unit area (OD/mm^2^). Blue lines across each graph are cutoff values. Cohort 1: (a) normal versus adenoma; (b) normal versus CRC. Cohort 2: (c) normal versus adenoma; (d) normal versus CRC.

In determining the specificity and sensitivity of the CD24 test and its ability to discriminate between patients with CRC or adenoma from individuals without endoscopic findings, cutoff values for the detection of adenomas and CRC were derived. The cutoff point for adenoma detection was 4118.87 (OD/mm^2^) and 8645.54 (OD/mm^2^) for CRC. The distributions of adenoma and CRC compared to normal colonoscopy cases are presented in Figures 2(a) and 2(b).

The sensitivity and specificity of the CD24 test for distinguishing CRC from normal subjects were 70.5% (95% CI, 54.8–83.2%) and 83.8% (95% CI, 60.4–96.4%), respectively. The CD24 test has a sensitivity of 84.2% (95% CI, 60.4–96.4%) for the detection of advanced adenomas with a specificity of 73.5% (95% CI, 61.4–83.5%). The positive predictive value (PPV) and negative predictive value (NPV) of CD24 for the detection of adenomas were 47.1% and 84.3%, respectively, versus 75.6% (PPV) and 81.7% (NPV) for CRC detection.

The second case-study cohort was comprised of 149 eligible subjects. Of them, 37 (24.8%) had adenomas, 35 (23.5%) had CRC, and 77 (51.7%) had normal colonoscopic examinations. In this refined group which excludes subjects with high CRP and WBC levels, improved values were obtained. The median values for CD24 expression were 15660 ± 762 (OD/mm^2^  ±  S.E.) in CRC patients, 16275 ± 1008 (OD/mm^2^  ±  S.E.) in patients with adenomas, and 7330 ± 741 (OD/mm^2^  ±  S.E.) in normal subjects. The median values for CD24 expression in lymphoid aggregates and inflammatory polyps were very low, similar to the first case series, 3143 ± 655 (OD/mm^2^  ±  S.E.).

The cutoff points for the detection of adenomas were 10289.3 (OD/mm^2^) and for CRC were 12405 (OD/mm^2^). The sensitivity of CD24 for the detection of CRC was 80.0% (95% CI, 63.1–91.6%) and for adenoma was 89.2% (95% CI, 74.6–97%), with a similar specificity to that obtained in the previously analyzed study group, 75.3% (95% CI, 64.2–84.4%) and 71.4% (95% CI, 60.0–97.0%) for CRC and adenoma, respectively (Table 2 and Figures 2(c) and 2(d)). The PPV for the detection of adenomas was 60.0% (95% CI, 45.9–73.0%); the NPV was 93.2% (95% CI, 83.5–98.1%). For CD24 in CRC the PPV and NPV were 59.6% (95% CI, 44.3–73.6%) and 89.2% (95% CI, 79.1–95.6%), respectively. Results of sensitivity and specificity for the two cohorts are summarized in Table 2.

**Figure 3:**
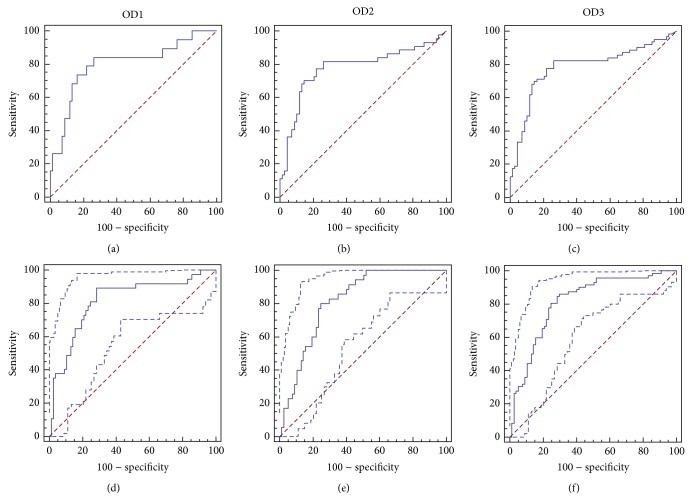
Empirical ROC curves. Cohort 1: (a) normal versus adenoma; (b) normal versus CRC; (c) normal versus adenoma and CRC. Cohort 2: (d) normal versus adenoma; (e) normal versus CRC; (f) normal versus adenoma and CRC.

The ROC curves with corresponding AUCs for both cohorts are shown in Figure 3. The AUC for adenoma and CRC detection was comparable at 0.80 and 0.78, respectively. The ROC curves in the second study showed a higher AUC of 0.815 compared to the first study.

The results obtained from the first cohort, when independent, blinded analysis was externally conducted, were varied. The specificity of CD24 for distinguishing between normal and CRC was lower at 65.0% (versus 83.8%) but comparable for adenoma, 76.7% versus 73.5% (data not shown). Univariate analyses revealed that CD24 levels above the cutoff were independent of the subject's gender, age, size of adenoma, or stage of CRC (data not shown).

No significant correlations were found between the expression of CD24 and three of the* CD24* polymorphisms examined, P170, P1056, and P1527. In addition, we did not find any correlation between these* CD24* SNPs and the prevalence of either adenomas or CRC. Interestingly, our data showed that the A1626G polymorphism appears to be associated with a lower incidence of CR neoplasia, although the results were not significant (*P* = 0.09, OR 0.61, and 95% CI 0.35–1.08). We also found that the TG/*del* (P1527), destabilizing dinucleotide deletion, combined with the A1626G polymorphism in the 3′ UTR of the* CD24* gene are associated with significantly lower level of CD24 protein (*p* = 0.026). Within the adenoma patients in the second cohort, 4 out of 37 (10.8%) patients carried both SNPs and all four exhibited significantly lower level of CD24 protein. Within the CRC patients 5 out of 35 (14.3%) patients carried both SNPs but only one exhibited undetectable level of CD24 protein.

## 4. Discussion

Patients with early and advanced CR neoplasia express high levels of CD24 compared to individuals without such abnormalities. A simple blood test measuring CD24 expression in PBLs can reliably differentiate between individuals with and without CR neoplasia. This data supports our previous findings that CD24 expression is an early event in the multistep process of CRC carcinogenesis and may be a potential marker of CRC, as well as a therapeutic target [14].

The concept of utilizing PBLs for the detection of adenomatous polyps and CRC could facilitate the approach for mass screening and prevention strategies. Based on our experience, in a few hundred subjects, all screeners with a positive result went on without any hesitation and agreed to undergo colonoscopy. Hence, this test can significantly reduce the morbidity and mortality from CRC by (i) ability to detect precursor lesions (e.g., adenomas), (ii) improving the public's compliance with CRC screening, and (iii) improving the compliance to undergo colonoscopy in those who turned out to be positive.

The cutoff points between the two study groups varied and were higher in the second case-study cohort, thus likely reflecting a difference in the methods and the use of a purified anti-CD24 SWA11 mAb [14, 15, 17].

The biological function of CD24 is not clearly understood. Studies have shown that CD24 is a ligand for P-selectin [11], an interaction that could be important in the dissemination of tumor cells and a key factor in recruiting lymphocytes into neoplastic tissue. CD24 is expressed in a wide variety of peripheral blood cells, including activated T cells, B-lineage cells, mature granulocytes, macrophages, and dendritic cells [18, 19]. Early in CRC development, dendritic cells recognize a change in the growth of cells and convey altered proteins to the lymphatic system. The lymphocytes are programmed against these altered proteins and send “tumor infiltrating lymphocytes” against the abnormal cells of the developing tumor. While the mechanism(s) by which CD24 is upregulated in PBLs and its function is under current investigation, their understanding may help to further improve the sensitivity, specificity, and most importantly the NPV of the test.

Data supporting this concept has been previously described by Cui et al., who evaluated IGF2 loss of imprinting (LOI) in lymphocytes, as a potential marker of CRC risk [20]. Thus, they showed that IGF2 LOI in lymphocytes is found in about 30% of CRC patients but only in 10% of healthy individuals and that an LOI blood test might be of value for population screening.

Several limitations of this study deserve comment. Firstly, several studies have reported CD24 expression in various other malignant conditions, including B-cell lymphomas, gliomas, lung, breast, urothelial tumors, and pancreatic cancer [21–24]. How CD24 levels in PBLs compare in CR neoplasia and other malignant processes warrants further study. Secondly, “bias” often plagues studies related to molecular diagnostic markers and may be a limitation in this study as well. Bias can be introduced from a number of sources including the study population itself, the metabolic state of the subject prior to blood collection, and during the processing of serum, storage, and analysis [21]. We paid special attention to limiting these factors and demonstrated that the results were independent of a number of patient-related features (i.e., gender, age). A systematic approach was employed to ensure reproducibility in blood and serum handling and analysis. Additionally, the serum analyses were performed by blinded investigators who were not aware of the results from colonoscopy or the patient classification. Nevertheless, additional larger-scale, multicenter studies are needed to externally validate our methodological approach and the results of the present study. Of note, we are currently developing a screening method based on ELISA and flow cytometry for a possible widespread use of the test. Preliminary results demonstrated similar or even slightly better sensitivity and specificity.

We are not overly concerned about false-positive results, as colonoscopy is indicated in all subjects, above the age of 50. However, we are concerned regarding the possibility of false negative results. Previous studies have shown that genetic variances in the* CD24* gene may affect mRNA and protein stability, leading to protein degradation. Therefore, we speculated that some subjects with CR neoplasia with low CD24 expression may carry* CD24* genetic variants. No significant correlation between the expression of CD24 and three of the* CD24* SNPs (P170, P1056, and P1527) examined was found. However, an association between the P1626 polymorphism and a lower incidence of CRC was seen, suggesting that this SNP may confer protection against the risk and progression of the disease. Moreover, the combination of P1626 with P1527 was significantly associated with lower levels of CD24 protein. As pointed above, within the adenoma patients in the second cohort, 10.8% of the patients (4/37) carried these two SNPs and expressed very low levels of CD24. Biochemical analysis has indicated that the P1527 deletion leads to rapid decay of* CD24* mRNA, which should result in reduced synthesis of the CD24 protein [13]. Thus, the expression of destabilizing genetic variants may explain some of the false negative data.

It should be noted that, based on our results, there is a significant difference in the predictive values between the general population and the patients screened in our studies. For example, the PPV for CRC in the second cohort was 59.6% but, in an average risk population with a prevalence of 0.5–1%, the PPV is only 3.06% meaning that, in randomly selected screenees at an average risk, with a positive answer for CD24, about 3% of them have CRC.

The only current noninvasive testing modality available for CRC screening is stool testing. While randomized controlled studies have shown a 15–20% reduction in CRC mortality using FOBT, the test is limited by low sensitivity, and it cannot detect adenomas [25]. In evaluating any screening program it is important to also measure programmatic adherence and performance over time. Stool testing is ineffective if it is not done every year over at least three consecutive years. In newer fecal DNA testing, higher sensitivities have been attained at a range of 71–91% [26]. In a recent meta-analysis the accuracy of fecal immunochemical tests for CRC was evaluated concluding that these tests are moderately sensitive and highly specific and have high overall diagnostic accuracy for detecting CRC depending on the cutoff values for positive test results [27]. However, it is presently not known what proportion of advanced adenomas is identified by fecal DNA testing and adenomatous polyp detection has been as low as 28–86%. The sensitivity and specificity of CD24 for distinguishing adenomas from normal subjects in our first trial were 84.2% (95% CI, 60.4–96.4%) and 73.5% (95% CI, 61.4–83.5%), respectively, whereas in the second trial a higher sensitivity of 89.2% (95% CI, 74.6–97%) was achieved for adenomas. Thus, it seems that the sensitivity of CD24 versus FIT is comparable in detecting cancer; however, it is much more sensitive than FIT for adenoma detection.

It is important to recognize that the variable results assessing the sensitivity of fecal DNA testing to date may be related to its evolving technology as improvements continue to be made. Newer fecal DNA tests, whether they involve technological advances made on existing tests or are new variants, warrant careful evaluation in future studies assessing screening cohorts.

There is undoubtedly a need for continued investigation of noninvasive means to identify neoplastic precursors related to CRC and more recent attention has focused on serum profiling. To date the only blood markers associated with CRC are the carcinoembryonic antigen (CEA) and SEPT9 [5, 6, 27], which have proven some prognostic utility. CEA is not suitable for screening as its sensitivity is only 30–40% for early CRC and, along with the low prevalence of CRC in unselected populations, the positive predictive value of CEA is unacceptably low and thus of little value in screening healthy subjects [28]. This also illustrates similar limitations of a number of other serum markers, including CA19-9, CA242, CA72-4, tissue polypeptide antigen (TPA), or tissue polypeptide specific-antigen (TPS) for the early detection of CRC [29]. SEPT9 in plasma, on the other hand, displays a high degree of sensitivity and sensitivity, making it a better method to detect CRC than guaiac-based FOBT and CEA but it is still very limited for the detection of adenomas [5–7].

In conclusion, our study identifies a potential serologic marker, CD24, for the detection of early CR neoplasia. The sensitivity and specificity of the CD24 test for distinguishing adenomas and CRC from subjects without findings on colonoscopy in this study were relatively high as compared to other available screening tests. CD24 alone, or in a panel of serologic markers, may be a very useful clinical tool in screening asymptomatic individuals for CR neoplasia and would better guide the utilization of resources for colonoscopic evaluation.

## 5. Conclusion

Colorectal cancer (CRC) frequently associates with high rates of mortality and morbidity, often resulting from late detection, underscoring the need for improved early detection, risk assessment, and intervention. Current available screening approaches are inadequate, and the development of accurate noninvasive biomarkers is needed.

The CD24 test involving its detection in peripheral blood leukocytes by serologic means holds promise for early detection and can successfully distinguish healthy subjects from patients with CRC with relatively high sensitivity and specificity as compared to other existing biomarkers.

A simple, noninvasive test that could reliably identify individuals with CR adenomas or early carcinoma not only would have great utility for the early detection of CRC, but also will be able to prevent the disease and be more widely accepted by the general population.

## Figures and Tables

**Table 1:** Sample population characteristics.

	Cohort 1 (*n* = 150)			Cohort 2 (*n* = 149)		
	Adenoma (*n* = 19)	Normal (*n* = 68)	CRC (*n* = 37)	Adenoma (*n* = 35)	Normal (*n* = 77)	CRC (*n* = 63)
Male	14	34	25	23	43	21
Female	5	34	12	12	34	42
Average age (y) ± S.E.	59.9 ± 13.00	55.8 ± 14.26	63.21 ± 10.68	63.72 ± 10.47	54.5 ± 6.73	62.7 ± 11.43

**Table 2:** Sensitivity and specificity values.

	Cohort 1		Cohort 2	
	Sensitivity (%)	Specificity (%)	Sensitivity (%)	Specificity (%)
Normal versus adenoma	84.2	73.5	89.2	71.4
Normal versus CRC	70.5	83.8	80.0	75.3
Normal versus adenoma and CRC	82.5	73.5	86.1	70.1
